# Hyperoside Protects HK-2 Cells Against High Glucose-Induced Apoptosis and Inflammation *via* the miR-499a-5p/NRIP1 Pathway

**DOI:** 10.3389/pore.2021.629829

**Published:** 2021-04-14

**Authors:** Jingbo Zhou, Shu Zhang, Xinyi Sun, Yan Lou, Jiangyi Yu

**Affiliations:** ^1^ Department of Endocrinology, Affiliated Hospital of Nanjing University of Traditional Chinese Medicine, Nanjing, China; ^2^ Department of Endocrinology, Jiangsu Provincial Hospital of Traditional Chinese Medicine, Nanjing, China

**Keywords:** hyperoside, HK-2 cells, apoptosis, inflammation, molecular biology

## Abstract

Hyperoside, a flavonol glycoside, is derived from plants of the genera *Hypericum* and Crataegus. Recent studies have indicated the anti-apoptotic and anti-inflammatory roles of hyperoside. The present study was designed to measure the effects of hyperoside on high glucose (HG)-treated HK-2 cells. HK-2 is a human papillomavirus 16 transformed cell line and can be used as a model for normal tubular cell. Cell apoptosis was examined by TUNEL assays and flow cytometry analysis. Inflammatory response was detected by Enzyme linked immunosorbent assay kits. Western blotting was applied to detect protein levels of apoptosis-related genes and inflammatory cytokines. Mechanistical assays including luciferase reporter and RNA pull down assays were applied to detect the binding relationship between molecules. We identified that hyperoside protected HK-2 cells against HG-induced apoptosis and inflammation. Moreover, miR-499a-5p was upregulated by hyperoside in a dose dependent manner. MiR-499a-5p inhibition rescued the suppressive effects of hyperoside on apoptosis and inflammation of HG-treated HK-2 cells. Furthermore, miR-499a-5p targeted NRIP1 to inhibit its mRNA expression, and further suppressed its translation. NRIP1 was downregulated by hyperoside in a dose dependent manner. Finally, rescue assays indicated that miR-499a-5p inhibition rescued the protective effects of hyperoside on apoptosis and inflammatory response of HK-2 cells by NRIP1. In conclusion, our findings revealed that hyperoside alleviates HG-induced apoptosis and inflammatory response of HK-2 cells by the miR-499a-5p/NRIP1 axis.

## Introduction

Diabetic nephropathy (DN) is a common complication of type I or type II diabetes [1]. Approximately 30–40% of diabetic patients are estimated to develop into DN [2]. Control on blood sugar, blood pressure, blood lipids, and diet intervention are mainly therapeutic strategies for DN [3]. Renal tubular damage induced by high glucose (HG) promotes the occurrence and development of DN [4]. Therefore, a comprehensive understanding of HG stimulated renal tubular damage contributes to seek the effective agents for DN.

Accumulating studies have indicated the importance of apoptosis and inflammation during renal tubular cell injury [5]. Apoptosis can result in progressive loss of renal cell, and thereby induce tubular atrophy, glomerular sclerosis, and renal interstitial fibrosis [6–8]. B-cell lymphoma-2 (Bcl-2) ad B-cell lymphoma-2 Associated X (Bax) are key modulators of apoptosis belonging to the Bcl-2 family. Bcl-2 independently heterodimerizes with Bax to suppress cell death, and the BH4 domain within the N-terminal region of Bcl-2 is essential for its anti-apoptotic activity [9]. Abnormal inflammation can result in destroyed renal architecture, companied by increased levels of pro-inflammatory cytokines including interleukin (IL)-1β, IL-6 and decreased levels of anti-inflammatory cytokines including IL-10 [10, 11]. Therefore, therapeutic strategies that inhibit apoptosis and inflammatory response of renal tubular cells are urgently needed.

Plant-derived herbal products have been found to potentially reduce apoptosis and inflammation of renal tubular epithelial cells. Hyperoside (quercetin-3-O-galactoside) is a flavonol glycoside derived from plants of the genera *Hypericum* and Crataegus [12]. Previous investigations have indicated that hyperoside plays an anti-inflammatory role, for example, hyperoside reduces the anti-inflammatory activities in mouse peritoneal macrophages by inactivation of nuclear factor-κB [13]. Hyperoside alleviates HG-induced inflammation in human umbilical vein endothelial cells [14]. Hyperoside is a putative therapeutic drug for vascular inflammatory diseases by inactivation of the high mobility group box 1 pathway [15]. In addition, hyperoside exerts protective effect on rats with heart failure via suppression of myocardial apoptosis [16]. Hyperoside alleviates ischemia-reperfusion induced tubular cell apoptosis [17].

HG-stimulated human renal proximal tubule (HK-2) cells were widely used as the *in vitro* models of DN [18–20]. The present study was designed to investigate the effects of hyperoside on apoptosis and inflammation of HG-treated HK-2 cells. In addition, the putative downstream pathway of hyperoside was explored.

## Materials and Methods

### Bioinformatics Analysis

The targets of miR-499a-5p were predicted by starBase [21] under the parameters of medium stringency in Degradome data, strict stringency in CLIP data, 5 programs in program number.

### Cell Culture and Treatment

Human renal proximal tubule (HK-2) cells and renal cell adenocarcinoma cells (786-O) were commercially provided by the American Type Culture Collection (ATCC, Rockville, IN, United States). The HK-2 cells were cultured under a 5% CO_2_ atmosphere at 37°C in Dulbecco’s modified Eagle medium (DMEM) containing% fetal bovine serum (FBS), streptomycin (100 mg/ml) and penicillin (100 units/mL, 1 ml). Upon reaching 60% confluence rate, cells were conducted to serum-starvation for 12 h. For HG treatment, HK-2 cells were cultured with 45 mmol/L of glucose for 24 h, while for control treatment, cells were cultured with 5.5 mmol/L of glucose for 24 h. Hyperoside at concentrations of 0, 10, 50, and 100 μmol/L were applied to treat HK-2 cells at room temperature for 6 h.

### Cell Transfection

The short hairpin RNA targeting nuclear receptor-interacting protein 1 (NRIP1), termed sh-NRIP1, miR-499a-5p mimics and NC mimics were provided by Biomics Biotechnologies (Jiangsu, China). Before transfection, treated HK-2 cells (5 × 10^5^ cells/well) were inoculated into 6-well plates. Next, cells were transiently transfected with these oligonucleotides using Lipofectamine 3000 (Invitrogen, CA, United States). Forty-eight hours after transfection, cells were harvested.

### Reverse Transcription-Quantitative Polymerase Chain Reaction (RT-qPCR)

Total RNA was extracted from treated HK-2 cells using the TRIzol reagent (Invitrogen). MiR-499a-5p was reverse transcribed into cDNA using a miRNA reverse transcription kit (Thermo Fisher Scientific). For NRIP1 reverse transcription, a PrimeScript reverse transcription reagent kit (Takara, Dalian, China) was applied. qPCR reactions were conducted on an ABI Prism 7500 RT PCR instrument (Applied Biosystems) using SYBR Premix Ex Taq (Takara). The thermocycling conditions were as follows: 50°C for 2 min, followed by 40 cycles of 95°C for 15 s and 60°C for 1 min. 2^−ΔΔ^C_t_ method [22] was utilized to analyze relative expression of miR-499a-5p or NRIP1 normalized to U6 or glyceraldehyde-3-phosphate dehydrogenase (GAPDH). Primer sequences for RT-qPCR are shown in Table 1.

**TABLE 1:** Primer sequences for RT-qPCR.

Relative primer sequences	
Molecules	Sequences
miR-499a-5p	Forward: 5′-GCC​CTG​TCC​CCT​GTG​CCT​T-3′
	Reverse: 5′-AAA​CAT​CAC​TGC​AAG​TCT​T-3′
U6	Forward: 5′-TGC​GGG​TGC​TCG​CTT​CGC​AGC-3′
	Reverse: 5′-CCA​GTG​CAG​GGT​CCG​AGG​T-3′
NRIP1	Forward: 5′-GCT​GGG​CAT​AAT​GAA​GAG​GA-3′
	Reverse: 5′-CAAAGAGGCCAGT AATGTGCTATC-3′
GAPDH	Forward: 5′-AGG​TCG​GTG​TGA​ACG​GAT​TTG-3′
	Reverse: 5′-TGT​AGA​CCA​TGT​AGT​TGA​GGT​CA-3′

### Cell Counting Kit-8 (CCK-8) Assay

A CCK-8 assay was used to measure cell viability. HK-2 cells (5 × 10^3^ cell/well) in different groups: control, HG, HG + 1 μmol/L of hyperoside, HG + 10 μmol/L of hyperoside, HG + 50 μmol/L of hyperoside, and HG + 100 μmol/L of hyperoside were inoculated into 96-well plate. After incubation, 10 μL of CCK-8 solution (Dojindo, Kyushu, Japan) was added to culture plate and cells were incubated for another 1 h at 37°C. Optical density of each well was evaluated by a Microplate Reader at wavelength of 450 nm.

### Terminal Deoxynucleotidyl Transferase dUTP Nick End Labeling (TUNEL) Assay

Cell apoptosis was investigated by a TUNEL assay kit. Treated HK-2 cells (4 × 10^3^ cell/well) were seeded on chamber slides. After 48 h of incubation, HK-2 cells were fixed in 4% paraformaldehyde, permeabilized with 0.1% Triton X-100 in PBS, and cultured with TUNEL reagents (Millipore; Merck KGaA, Darmstadt, Germany). TUNEL positive stained cells were observed by an optical microscope (Olympus, Tokyo, Japan).

### Flow Cytometry Analysis

An apoptosis kit (Solarbio, Beijing, China) was used to measure apoptosis. HK-2 cells by different treatments were transferred to for. Cells were washed and resuspended in 100 μL of 1 × Binding Buffer after 5 min of centrifugation in a centrifuge tube. Next, 5 μL of Annexin V-FITC solution was added to the cells and cells were incubated at 37°C for 15 min in the dark. Afterward, cells were washed again and re-suspended in 200 μL of 1 × Binding Buffer, and 5 μL of PI solution was added. Cell apoptosis rate was analyzed by flow cytometry within 1–2 h.

### Western Blot

Western blotting was conducted according to a previous study [23]. The primary antibodies used in the study are listed as follows: anti-Bax (ab32503, 1/1000), anti-Bcl-2 (ab32124, 1/1000), anti-IL-10 (ab133575, 1/2000), anti-IL-6 (ab233706, 1/1000), anti-IL-1β (ab234437, 1/1000), and anti-GAPDH (ab9485, 1/2500).

### Enzyme Linked Immunosorbent Assay (ELISA)

HK-2 cells by different treatments were centrifuged at 1,000 × *g* for 20 min. The levels of IL-10, IL-6 and IL-1β in supernatants were examined by corresponding ELISA kits (ab185986, ab178013, ab214025, Abcam). The absorbance values at 450 nm were detected using a microplate reader (BioTek Instruments).

### Luciferase Reporter Assay

Binding sequences between miR-499a-5p and NRIP1 3′ untranslated region (UTR) were predicted from starBase. The HK-2 cells were plated into 24 well plates and reached 80% confluency. Next, wide-type (Wt) and mutant-type (Mut) NRIP1 3′ UTR were inserted into pmirGLO vectors (Invitrogen) to generate pmirGLO-NRIP1-Wt and pmirGLO-NRIP1-Mut plasmids. Afterward, these vectors were cotransfected with miR-499a-5p inhibitor or NC inhibitor into HK-2 cells. After 48 h, the luciferase activities were measured using a Lucifer Reporter Assay System (Promega, Madison, WI, United States) and were normalized to Renilla luciferase activity.

### RNA Pull Down Assay

Biotin-labeled miR-499a-5p-Wt and miR-499a-5p-Mut were synthesized by RiboBio. The cells were treated with 50 nM of biotin-labeled NC, miR-499a-5p-Wt and miR-499a-5p-Mut for 48 h. Next, cells were rinsed by PBS, and cultured with RIPA lysis buffer for 10 min. Subsequently, cells were cultured at 4°C for 3 h. The beads were washed with precooled lysis buffer twice, low salt buffer 3 times and high salt buffer once. TRIzol was used to purify the binding RNA. Finally, RT-qPCR revealed the relative enrichment of NRIP1.

### Statistical Analysis

The data obtained from ≥3 independent experiments are shown as mean ± the standard deviation. Statistical significance of difference was analyzed using Student's t-test (for comparisons between 2 groups) and analysis of variance followed by Tukey’s *post hoc* test (for comparisons more than 2 groups). *p*-values less than 0.05 were statistically significant. Statistical analyses were conducted using SPSS 22.0 (IBM, Armonk, NY, United States).

## Results

### Hyperoside Protects HK-2 Cells Against HG-Induced Apoptosis and Inflammation

**FIGURE 1:**
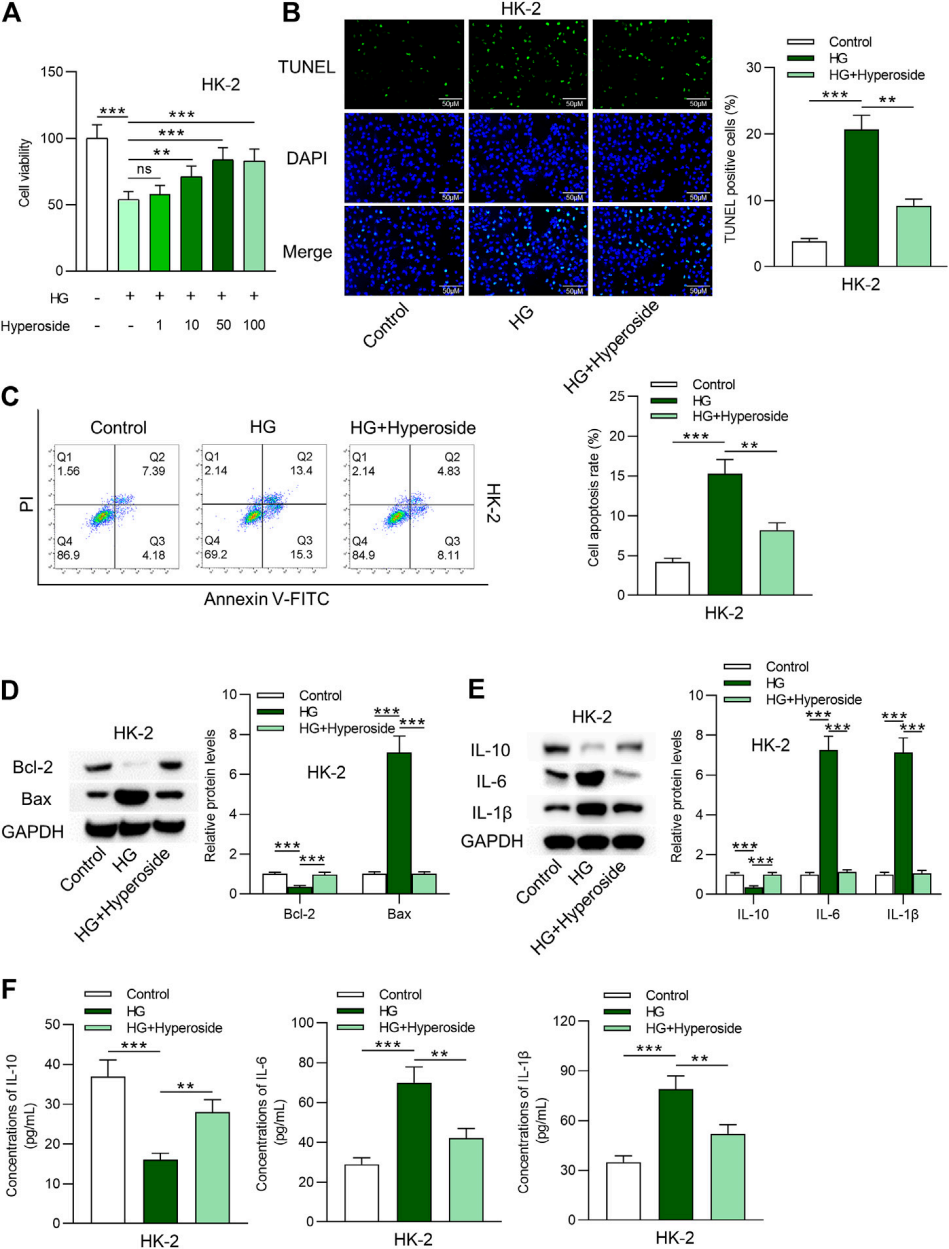
Hyperoside protects HK-2 cells against HG-induced apoptosis and inflammation. **(A)** Viability of HK-2 cells by different treatments was measured by CCK-8 assay. **(B, C)** TUNEL and FITC/PI staining assays revealed apoptosis of HK-2 cells by different treatments: control; HG; HG + hyperoside. **(D)** Bcl-2 and Bax proteins were detected using western blotting performed on extracts of HK-2 cells. **(E)** Influence of hyperoside on IL-10, IL-6 and IL-1β proteins in HG induced HK-2 cells. **(F)** ELISA revealed IL-10, IL-6 and IL-1β contents in HK-2 cells by different treatments: control; HG; HG + hyperoside. **p < 0.01, ***p < 0.001, ns indicates no significance.

First, viability of HK-2 cells under control condition, HG condition, and HG combined hyperoside condition was measured. As revealed in Figure 1A, cell viability was reduced by HG treatment. The inhibitory effects of HG on cell viability were rescued by hyperoside in a dose dependent way. We used 50 nm of hyperoside in the following assays. Data from TUNEL assays **(** Figure 1B) and flow cytometry analysis (Figure 1C **)** indicated that cell apoptosis was induced by HG treatment and was reduced by hyperoside. Western blot analysis revealed that increased Bax expression and decreased Bcl-2 expression induced by HG were rescued by hyperoside (Figure 1D). Furthermore, protein levels of IL-10, IL-6 and IL-1β in HG-treated HK-2 cells were measured. HG-induced upregulation of IL-6 and IL-1β protein levels and downregulation of IL-10 protein levels, and hyperoside rescued the influence of HG treatment on IL-10, IL-6 and IL-1β (Figure 1E). Finally, concentrations of these proinflammatory cytokines were detected by ELISA kits. The results indicated that hyperoside reversed the stimulating influence of HG on inflammatory response of HK-2 cells (Figure 1F).

### Hyperoside alleviates HG-induced apoptosis and inflammation by upregulation of miR-499a-5p

**FIGURE 2:**
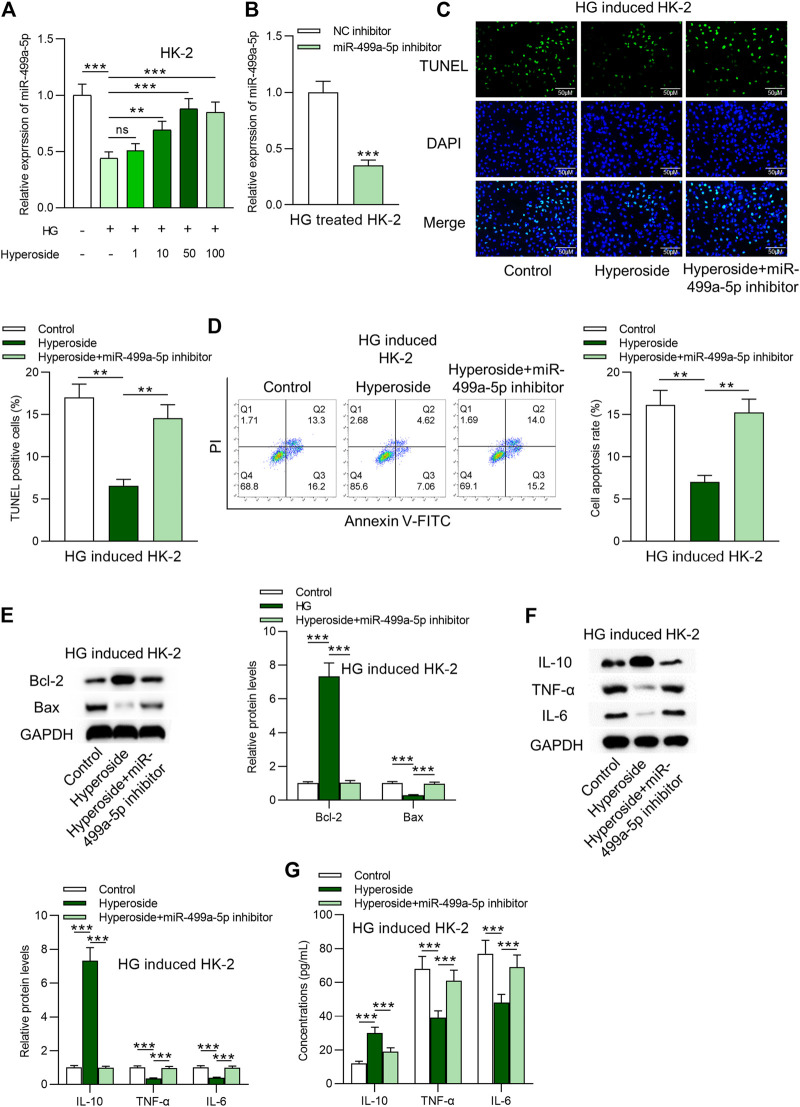
Hyperoside alleviates HG-induced apoptosis and inflammation by upregulation of miR-499a-5p. **(A)** RT-qPCR was performed on extracts of HK-2 cells under different treatments: control; HG; HG + hyperoside to detect miR-499a-5p expression. **(B)** Knockdown efficiency of miR-499a-5p in HG-treated HK-2 cells was evaluated by RT-qPCR. **(C,D)** TUNEL and FITC/PI staining assays were applied to detect influence of miR-499a-5p inhibitor on hyperoside in HG-treated HK-2 cells. **(E)** Western blotting revealed Bcl-2 and Bax proteins. **(F,G)** Western blotting and ELISA were performed to reveal IL-10, IL-6 and IL-1β levels. **p < 0.01, ***p < 0.001, ns indicates no significance.

Next, we identified that miR-499a-5p was downregulated by HG and was further dose-dependently upregulated by hyperoside in HK-2 cells (Figure 2A). RT-qPCR was applied to detect the knockdown efficacy of miR-499a-5p in HG treated HK-2 cells. Data in Figure 2B suggested that miR-499a-5p expression was effectively knocked down by miR-499a-5p inhibitor. MiR-499a-5p inhibitor rescued the antiapoptotic effects of hyperoside on HG-treated HK-2 cells (Figures 2C–E). Results from Western blot analysis (Figure 2F) and ELISA (Figure 2G) showed that miR-499a-5p inhibition rescued the increase of IL-10 levels, and the decrease of IL-6 and IL-1β levels induced by hyperoside in HG treated HK-2 cells.

### MiR-499a-5p Targets NRIP1 in HK-2 Cells

**FIGURE 3:**
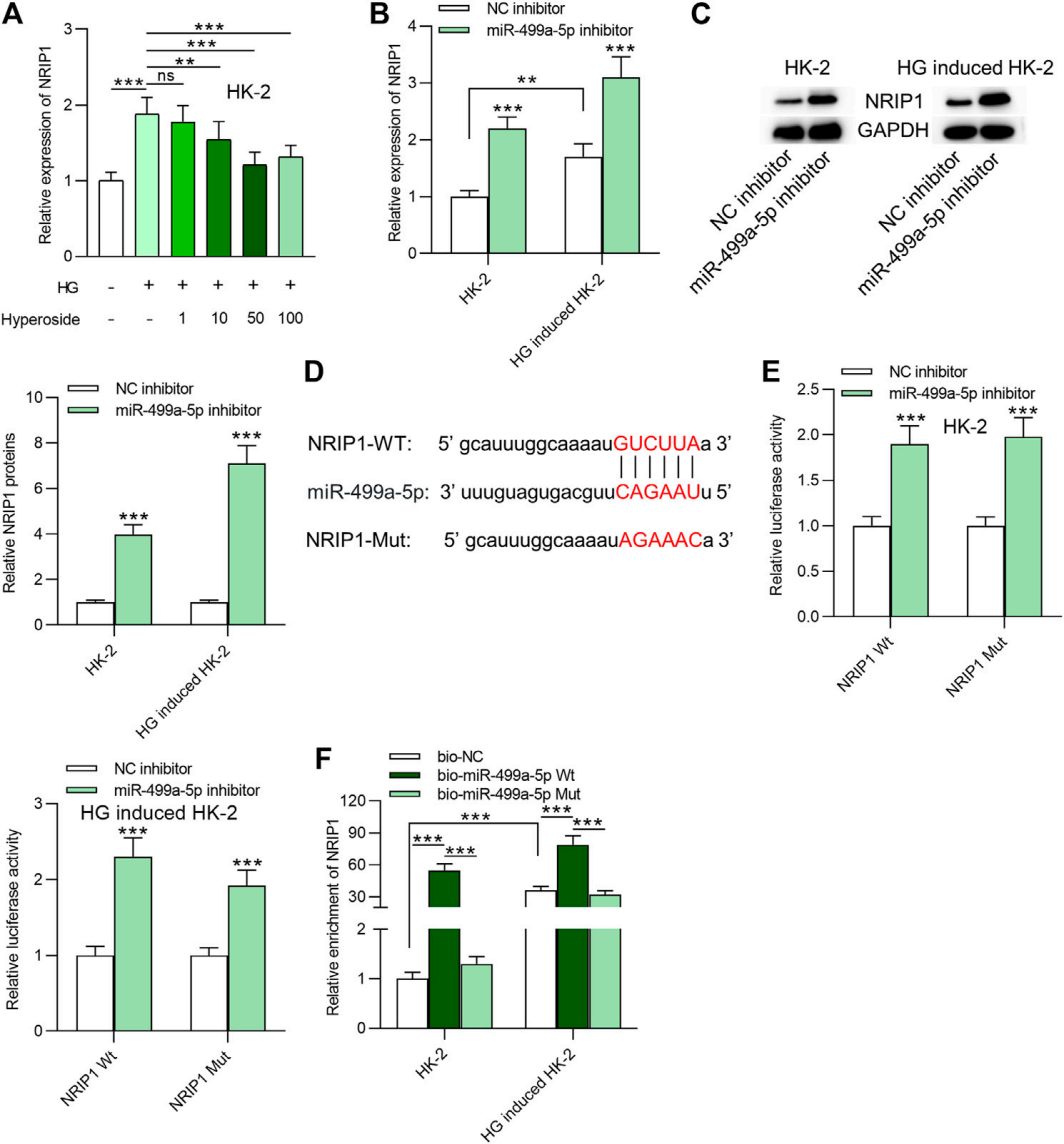
MiR-499a-5p targets NRIP1 in HK-2 cells. **(A)** RT-qPCR was performed on extracts of HK-2 cells under different treatments: control; HG; HG + hyperoside to detect expression of NRIP1. **(B, C)** The influence of miR-499a-5p inhibitor on NRIP1 mRNA and protein expression in HK-2 cells with or without HG treatment. **(D)** Binding site of miR-499a-5p and NRIP1 was predicted from starBase. **(E)** Luciferase activity of pmirGLO-NRIP1-Wt and pmirGLO-NRIP1-Mut plasmids in HK-2 cells transfected with miR-499a-5p inhibitor. **(F)** A RNA pull down assay was applied to detect relative enrichment of NRIP1. **p < 0.01, ***p < 0.001, ns indicates no significance.

NRIP1 was identified as a downstream target of miR-499a-5p based on starbase prediction. HG resulted in upregulation of NRIP1, and hyperoside treatment dose-dependently reduced its expression in HK-2 cells **(** Figure 3A). MiR-499a-5p inhibitor promoted NRIP1 mRNA and protein levels in HK-2 cells regardless of HG treatment (Figures 3B,C). The binding sequences of miR-499a-5p and NRIP1 were predicted from starBase and were shown in Figure 3D. Binding site of NRIP1 was mutated for the next luciferase reporter assay. As indicated in Figure 3E, transfection of miR-499a-5p inhibitor enhanced the luciferase activity of pmirGLO-NRIP1-Wt plasmids and had no significance influence on that of pmirGLO-NRIP1-Mut plasmids in HK-2 cells regardless of HG treatment. Moreover, data of RNA pulldown assay showed that NRIP1 was abundantly enriched in products pulled down by bio-miR-499a-5p Wt, compared with that pulled down by bio-miR-499a-5p Mut, indicating the interaction of miR-499a-5p and NRIP1 **(** Figure 3F).

### Hyperoside Suppresses Apoptosis and Inflammatory Response in HG-Treated HK-2 Cells via the miR-499a-5p/NRIP1 Pathway

**FIGURE 4:**
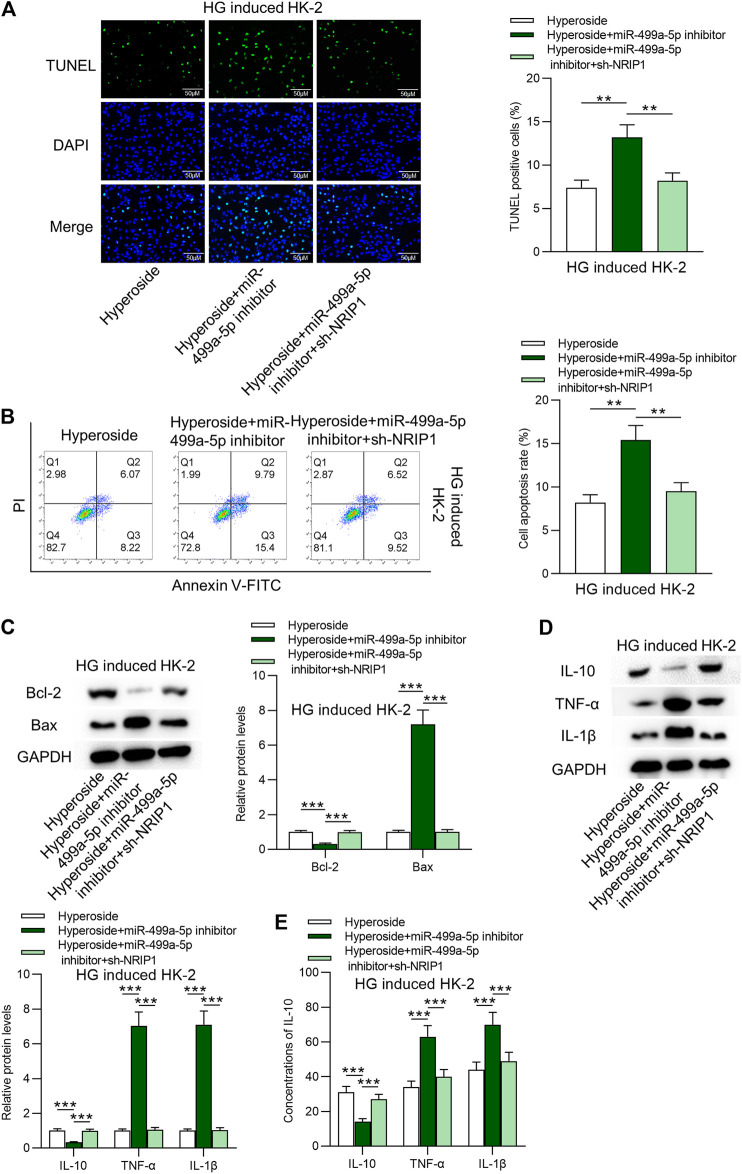
Hyperoside suppresses apoptosis and inflammation of HG-treated HK-2 cells via the miR-499a-5p/NRIP1 pathway. **(A, B)** TUNEL and FITC/PI staining assays revealed apoptosis of HG-treated HK-2 cells by different treatments: hyperoside; hyperoside + miR-499a-5p inhibitor; hyperoside + miR-499a-5p inhibitor + sh-NRIP1. **(C)** Western blotting performed on extracts of HK-2 cells was conducted to reveal Bcl-2 and Bax proteins. **(D, E)** Western blotting and ELISA were performed to reveal IL-10, IL-6 and IL-1β levels. **p < 0.01, ***p < 0.001.

Finally, the rescue assays were conducted. Figures 4A–C revealed that NRIP1 inhibition rescued the proapoptotic influences of miR-499a-5p on HK-2 cells. The stimulating effects of silenced miR-499a-5p on protein levels of IL-10, IL-6 and IL-1β were rescued by downregulation of NRIP1 (Figures 4D,E). Hyperoside induced apoptosis of 786–0 cells, while neither miR-499a-5p nor NRIP1 had rescue effects on hyperoside induced apoptosis of 786–0 cells (Supplementary Figures S1A,B).

## Discussion

Our findings revealed that HG treatment induced apoptosis and inflammatory response of HK-2 cells, and hyperoside protected HK-2 cells against HG induced apoptosis and inflammation. MiR-499a-5p is downregulated by HG treatment in HK-2 cells. Hyperoside can induce the upregulation of miR-499a-5p. Hyperoside inhibited apoptosis and inflammation of HG treated HK-2 cells by upregulation of miR-499a-5p. Furthermore, miR-499a-5p targeted NRIP1 3′UTR to inhibit its mRNA expression, and further reduce its translation.

MiRNAs play significant roles in apoptosis and inflammatory response of HK-2 cells, for example, Arbutin upregulates miR-27a to inhibit apoptosis of HK-2 cells stimulated by HG [24]. MiR-34b targets IL-6R to alleviate HG-stimulated inflammatory response and apoptosis of HK-2 Cells [25]. MiR-455-3p decreased ROCK2 to reduce inflammatory cytokine levels in HG stimulated HK-2 cells [26]. In the present study, HG treatment induced the low expression of miR-499a-5p in HK-2 cells, and hyperoside rescued the downregulation of miR-499a-5p caused by HG in HK-2 cells. Inhibition of miR-499a-5p rescued the protective effects of hyperoside on apoptosis and inflammation of HG treated HK-2 cells. MiR-499a-5p expression has been found to be decreased in the livers of mice fed a high-fat diet, and is associated with the insulin signaling pathway and glycogen synthesis [27]. Levels of miR-499-5p are reduced in erythrocytes of African American pre-diabetic patients [28]. A similar expression pattern of miR-499-5p is also found in patients with DN or diabetic polyneuropathy [29]. In addition, miR-499-5p is downregulated among patients with diabetic end-stage renal disease [30].

Subsequently, NRIP1 was verified as the downstream target of miR-499a-5p. NRIP1 inhibition rescued the effects of miR-499a-5p inhibitor on hyperoside in HG-stimulated HK-2 cells, indicating the putative role of NRIP1 in DN. NRIP1 is also named as receptor-interacting protein 140 (RIP140). The RIP140 protein functions as a coactivator or a corepressor following its recruitment to target genes [31], and is essential in glucose metabolism [32]. Xue et al. have revealed that NRIP1 is associated with subclinical inflammation in patients with type 2 diabetes [33]. RIP140 was shown to induce the expression of proinflammatory factors including IL-6, and IL-1β [34]. RIP140 degradation facilitates the alteration of activity of several proinflammatory cytokines in endotoxin tolerance [35]. RIP140 has the proinflammatory potential in response to alteration of the intracellular cholesterol status in macrophages [36]. Moreover, RIP140 was found to exert regulatory functions in acute and chronic inflammatory diseases [37]. Hyperoside has been reported to induce apoptosis of 786-O renal cancer cells by miR-27a [38], indicating the important role of botanicals and their derivates in the anticancer activity. The present study revealed that 786-O cell apoptosis was promoted by hyperoside. Neither miR-499a-5p nor NRIP1 had rescue effects on hyperoside induced apoptosis of 786-0 cells.

In conclusion, our findings innovatively revealed that hyperoside protected HK-2 cells against HG-stimulated apoptosis and inflammation via the miR-499a-5p/NRIP1 axis (Supplementary Figure S2). However, this study only focused on *in vitro* model, which is a limitation of the present study. Our future research will focus on the *in vivo* model systems. Furthermore, HK-2 is a human papillomavirus 16 transformed cell line and is used as a model as normal tubular cell with limitations, which reacts differently to primary epithelial cells.

## Data Availability

The datasets used during the current study are available from the corresponding author on reasonable request.
