# Supplementary figures and images for: Hyperoside Protects HK-2 Cells Against High Glucose-Induced Apoptosis and Inflammation via the miR-499a-5p/NRIP1 Pathway
Source: Pathol Oncol Res. 2021 Apr 14;27:629829. doi: 10.3389/pore.2021.629829 (PMC8262192; doi:10.3389/pore.2021.629829)

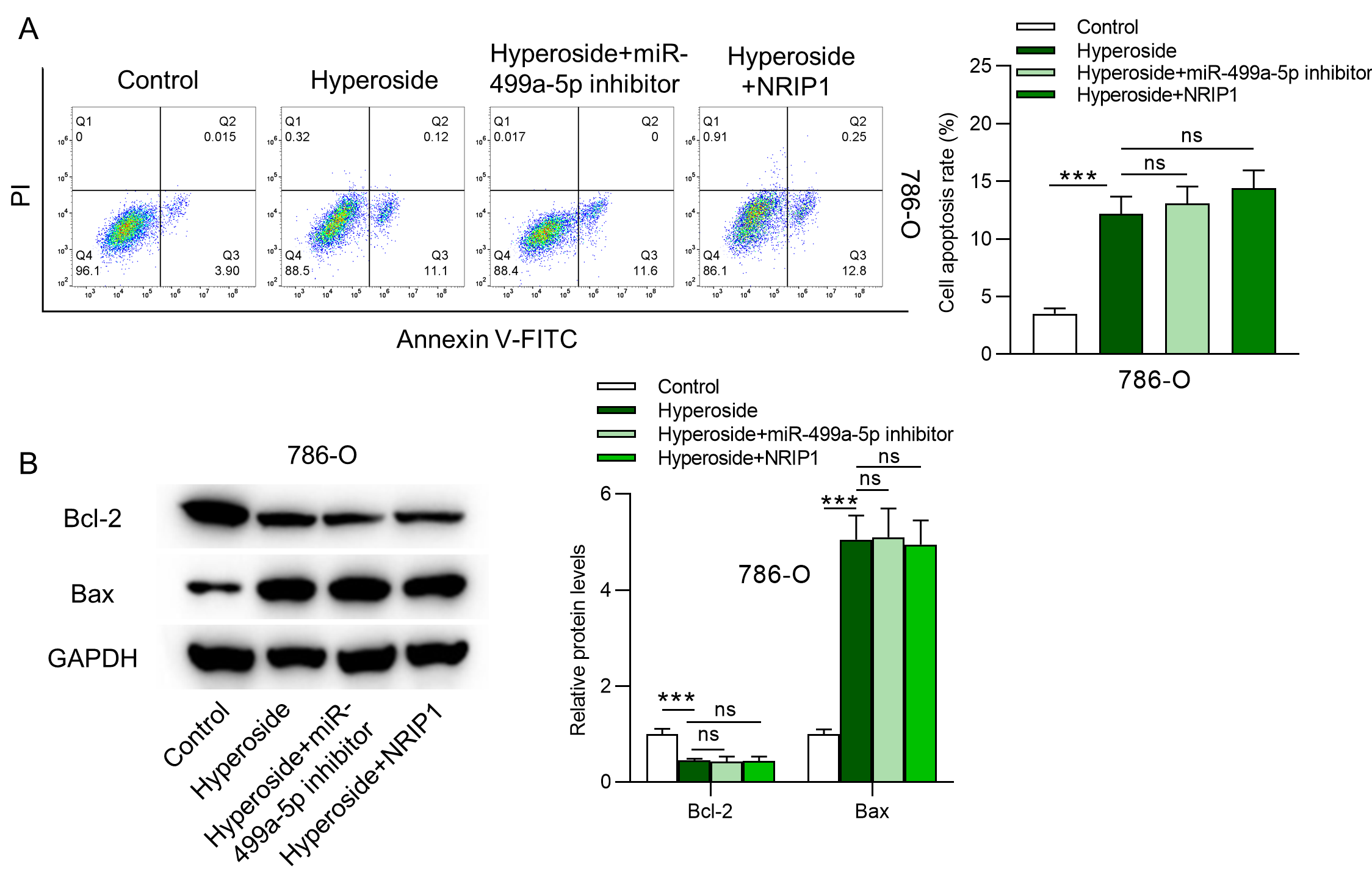

Supplement: Supplementary file 1 [file Image1.TIF]

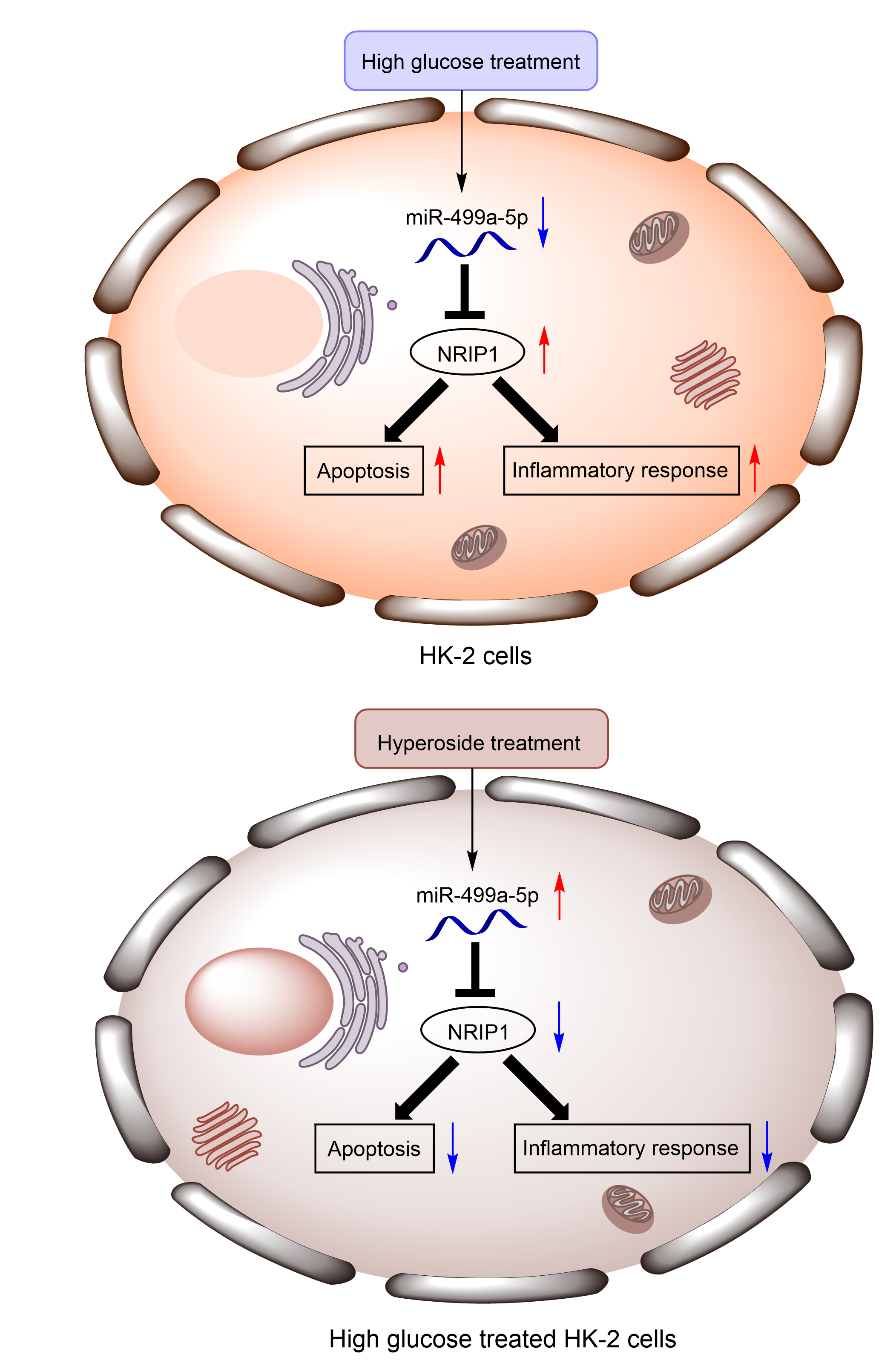

Supplement: Supplementary file 2 [file Image2.TIF]
